# Knowledge of Vulvovaginal Candidiasis Characteristics, Signs, Symptoms, and Appropriate Treatment Among Portuguese Pharmacy Professionals

**DOI:** 10.3390/healthcare13040402

**Published:** 2025-02-13

**Authors:** Tiago Oliveira, Ângelo Jesus, João P. Martins, Patrícia Correia, Fernando Moreira

**Affiliations:** 1 Escola Superior de Saúde, Instituto Politécnico do Porto, Rua Dr. António Bernardino de Almeida, 4200-072 Porto, Portugal; tiago_sousa_oliveira@hotmail.com (T.O.); acj@ess.ipp.pt (Â.J.); jom@ess.ipp.pt (J.P.M.); pcorreia@ess.ipp.pt (P.C.); 2 LAQV/REQUIMTE, Escola Superior de Saúde, Instituto Politécnico do Porto, Rua Dr. António Bernardino de Almeida, 4200-072 Porto, Portugal; 3 CEAUL—Centro de Estatística e Aplicações, Faculdade de Ciências, Universidade de Lisboa, 1749-016 Lisboa, Portugal

**Keywords:** vulvovaginal candidiasis, vaginitis, clotrimazole, fluconazole, antifungals, pharmacy

## Abstract

Background/Objectives: Vulvovaginal candidiasis (VVC) is a common cause of vaginitis. Over-the-counter drugs are usually dispensed by pharmacy professionals to treat this condition without prior medical consultation. This study aimed at assessing the knowledge of Portuguese pharmacy professionals regarding VVC. Methods: An online questionnaire including questions about the symptoms and treatment of VVC was distributed between March and April of 2024. Results: A total of 376 professionals participated in this study. There was a significantly lower proportion of men (*p* = 0.007) and pharmacy technicians (*p* = 0.005) who correctly identified the main causative agent of VVC when compared to women and pharmacists. Only 30% of women correctly identified the number of VVC episodes in the same year they classified as complicated, but this was significantly higher (*p* = 0.038) than the proportion of men who identified complicated VVC (20%). Topical clotrimazole preparations were the more frequently identified medicines for the treatment of uncomplicated VVC, and fluconazole-containing medicines were the preferred choice for the treatment of complicated VVC. Conclusions: This study highlights the need to improve education and training for pharmacy professionals. By addressing these knowledge gaps, pharmacists and pharmacy technicians can provide more accurate and effective advice to patients.

## 1. Introduction

Vulvovaginal candidiasis (VVC) can be defined as the occurrence of signs and symptoms of inflammation in the presence of *Candida* species in the absence of other infectious agents [1]. *C. albicans* is the most common infectious agent, followed by different *Candida* species (including *Candida glabrata, Candida tropicalis, Candida parapsilosis,* and *Candida guilliermondii*) [2]. While systematic reviews from the 2000s pointed to bacterial vaginosis as the leading cause of vaginitis [3], recent studies examining vaginal samples of 470 women have found VVC to be the primary cause of vaginitis among Portuguese women (74.4% of the participants with vulvovaginitis) [4]. In the study by Fernandes et al. [4], a high incidence of colonization with *Candida* spp. among asymptomatic women was reported (63.7%), indicating that the Portuguese population is at a heightened risk of developing VVC. Still, the epidemiological data reported are associated with high uncertainty, and under-reporting is very likely. This may be explained by the fact that it is not a reportable disease and over-the-counter (OTC) treatment is available. In addition, epidemiological studies carried out on this topic are often associated with different methods for diagnosing the infection and the use of non-representative samples [5,6]. It should be noted that the presence of *Candida* in the vagina is not synonymous with infection, as asymptomatic women can be colonized. At the same time, the signs and symptoms of the disease, although frequent, are non-specific and partly overlap with diseases such as trichomoniasis, bacterial vaginosis, and gonorrhea [3]. Typical clinical manifestations include vulvar itching and burning, along with vaginal discomfort that can result in painful intercourse and urination, often accompanied by redness, swelling, and cracks in the vulvar and vaginal areas [7]. Despite the high level of uncertainty, it is recognized that millions of women are affected by the disease worldwide every year, and 70–75% of women will have an episode of VVC in their lifetime [5]. The importance of early diagnosis and immediate treatment of VVC cannot be overlooked. Untreated VVC is associated with pelvic inflammatory disease, pelvic abscesses, infertility, miscarriages, and ectopic pregnancy [8]. It is also important to mention the impact of the disease on the mental health of the women affected, due to the pain, discomfort, anxiety, and altered emotional and sexual relationships caused by the disease [5]. VVC has been regularly divided in terms of classification into uncomplicated VVC and complicated VVC. Uncomplicated VVC might be defined by the sporadic occurrence of mild or moderate disease caused by *C. albicans* in immunocompetent women. On the other hand, complicated VVC includes more severe cases caused by non-*albicans Candida*, associated with pregnancy or other conditions such as uncontrolled diabetes or immunosuppression and recurrent VVC (at least four episodes of VVC per year) in immunosuppressed women [1,6,9,10].

Given the high incidence of genitourinary infections, several topical antifungals are marketed as OTC medicines [1,11]. It is important to acknowledge that, in Europe, one out of two packages of the medicines sold is a nonprescription product [12]. The gradual change in the legal status of various medicines from prescription to nonprescription has been noticeable for a long time [13]. These combined data suggest that the trend towards OTC availability will continue to increase. Pharmacies are amongst the preferred places to access health advice and care. Recent findings suggest that, in the USA, patients visit their community pharmacies almost twice as often as they visit their physicians or other healthcare professionals [14]. Pharmacies are known for their extended hours, extensive geographic reach, and the convenience of not requiring appointments [15,16]. This proximity to the community enables pharmacies to relieve pressure on the overwhelmed healthcare systems. The contribution of pharmacies to freeing up hospital services to deal with urgent patients is crucial and is particularly noticeable at the most critical times. The resolution of clinical situations that did not require a doctor for analysis and more specific medical tests was fundamental during the COVID-19 pandemic [17,18]. Pharmacy assistance through OTC counseling has a crucial role in the community, potentially leading to enhanced clinical outcomes and prudent use of OTC medicines [19,20,21]. In particular, regarding the treatment of VVC, it is acknowledged that both pharmacists and pharmacy technicians can assist patients in accessing the appropriate treatments [22]. However, while studies have examined physicians’ management of the disease [23,24], students’ and graduates’ awareness [25,26], or women’s knowledge on the topic [27,28,29,30,31], there is a lack of information on pharmacy technicians’ and pharmacists’ competence in recognizing symptoms and differentiating VVC from other vaginal infections. Similarly, the current literature does not adequately address pharmacy technicians’ and pharmacists’ familiarity with evidence-based treatment protocols for VVC, particularly for recurrent cases.

Differential diagnosis is considered adequate in identifying genitourinary fungal infections and, when properly conducted, allows for the selection of OTC. The widespread availability of these drugs allows for faster intervention for women who need them, while also relieving the pressure in hospital units. However, incorrect diagnosis of the pathology can result in non-rational use of the drugs, delay the correct intervention, and promote microbial resistance. Studies carried out in the 1990s and early 2000s suggested that the community was not adequately prepared to identify the signs and symptoms of fungal infections treatable with OTC medicine [27,32]. Pharmacy professionals responsible for dispensing OTC medicine can be expected to be familiarized and prepared to identify cases of genitourinary fungal infections that can be treated with these drugs. Nonetheless, some characteristics that overlap with parasitic and/or bacterial infections and the existence of so-called ‘complicated cases’ of fungal infection whose treatment with OTC drugs is debatable challenge professionals.

This study aims to assess pharmacy professionals’ knowledge: (i) of the signs and symptoms of VVC; (ii) of the differences between uncomplicated VVC and recurrent VVC; (iii) and of the treatments available for VVC.

## 2. Materials and Methods

To assess the knowledge level of pharmacy professionals working in Portugal, regarding the clinical characteristics and treatment of VVC, an online questionnaire was developed, based on the recommendations of the Portuguese Society of Gynecology [33], and was also corroborated by a prior literature review. Participants were approached via social media channels, including LinkedIn™, Instagram™, and Facebook™, where they were given the option to voluntarily access Microsoft Forms™ software and respond to a questionnaire. This questionnaire was previously analyzed by 30 people, and none of the participants identified any deficiencies in its wording, clarity, or ease of understanding. No overall statistical evaluation of the pre-test was performed. Data collection took place from 26 March 2024 to 22 April 2024.

Inclusion criteria encompassed professionals working in a Portuguese community or hospital pharmacies, while exclusion criteria were applied to professionals working outside of the country. Only those who completed the informed consent form provided with the questionnaire were included.

The findings were analyzed with the IBM™ Statistical Package for the Social Sciences (SPSS) version 29 for Windows.

Age, place of residence, and education level were among the variables considered to describe the sample. To measure participants’ knowledge about VVC, questions were asked about the etiological agent and symptoms of this condition and also the differences between severe and simple VVC (based on the number of episodes). Knowledge of potential pharmacological therapies was assessed using questions concerning OTC drugs used for VVC and which of the listed treatments would be recommended for complicated VVC. As potential response options, medications that are universally available in any pharmacy in Portugal were presented.

Categorical variables were described using counts and percentages. Quantitative variables were summarized through the median and the 25th and 75th percentiles.

Fisher’s exact test was used to assess knowledge regarding the main cause of vaginitis and its primary etiological agent across various social demographic factors. The Mann–Whitney test was applied to evaluate the effect of sex, profession, and high academic degree on the number of correct symptoms identified, the number of correct medications for treating VVC, the number of VVC episodes within the same year to determine if they qualified as complicated, and the number of correct medications for the recommended treatment of complicated VVC. Relationships between ordinal variables were analyzed using Spearman’s correlation. When differences were identified in two or more sociodemographic factors, multivariate analysis was conducted using logistic regression or linear regression, as applicable in each situation.

## 3. Results

Out of the 389 individuals who started to answer the questionnaire, 13 individuals chose to stop participating before finishing it, leading to a 96.7% completion rate (376 individuals). The demographic profile of the participants (Table 1) shows a significant majority of women (86.4%, 325 individuals), with most of them having one to five years of professional experience (41.2%, 155 participants). The majority held a master’s degree and PhD as their highest educational qualification (66.2%, 249 people), and pharmacists made up the largest professional group (66.0%, 248 individuals).

The majority of participants in this study identified VVC as the main cause of vaginal inflammation (57.7%, n = 217). No differences were detected in the responses based on the sociodemographic variables studied (Table 2).

More than 95% of participants (n = 358) recognized *C. albicans* as the main agent responsible for VVC. A small fraction (2.10%, eight individuals) indicated they were unsure, by selecting ’Does not know’, which was the second most common response, albeit significantly less frequent than the correct answer. Significant differences were detected in terms of the variables sex (*p* = 0.021) and profession (*p* = 0.048).

Both sexes identified *C. albicans* as the main agent, although a significantly higher percentage was observed in females (96.3%, 313 individuals) compared to males (88.2%, 45 individuals). Less than 1% of female participants (three individuals) identified *Candida glabrata* as the main etiological agent, whereas this response was given by nearly 8% (four individuals) of male participants.

Both professions recognized *C. albicans* as the main etiological agent of VVC, with a significantly greater proportion observed among pharmacists (97.2%, 241 individuals) than pharmacy technicians (91.4%, 117 individuals). While less than 1% of pharmacists (two individuals) indicated *Candida glabrata* as the main etiological agent, this response was selected by nearly 4% (five individuals) of pharmacy technicians.

Given the differences identified in terms of the variables sex and profession, a logistic regression model was adjusted, with the identification of the main etiological agent of VVC (*C. albicans*) as the dependent variable (Table 3).

**Figure 1:**
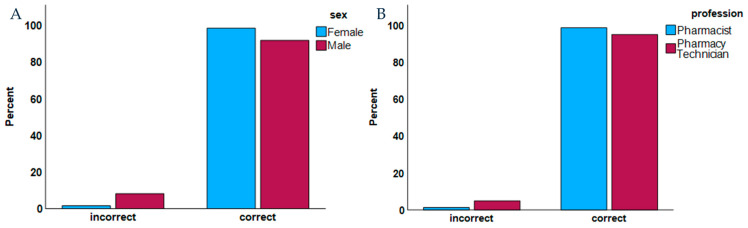
Bar plot of the ability to identify *Candida albicans* as the correct main etiological agent of VVC by (**A**) sex; (**B**) profession.

The multivariate analysis confirms that females (*p* = 0.007) and the pharmacist profession (*p* = 0.005) demonstrated greater knowledge regarding the main etiological agent of VVC (Table 4), although both sexes and professions exhibited high levels of knowledge (Figure 1).

**Figure 2:**
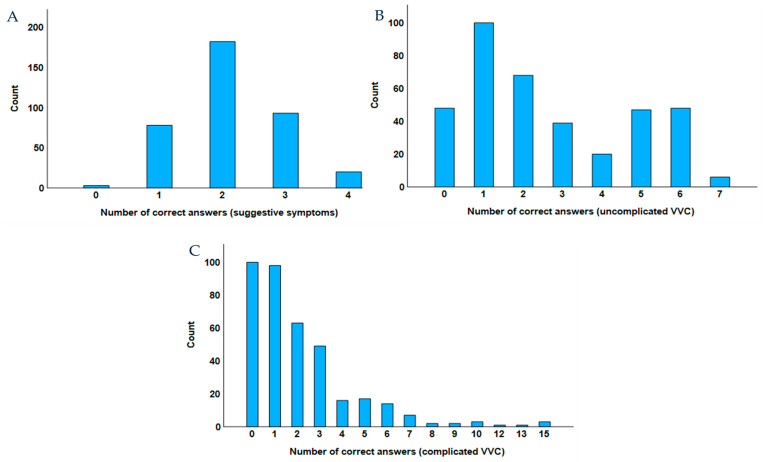
Bar plot of the number of correct answers of (**A**) suggestive symptoms; (**B**) uncomplicated VVC; and (**C**) complicated VVC.

Associated with the question ‘which of the following is a suggestive symptom for VVC?’, there were several possible choices. Four of the options were correct—‘Thick vaginal discharge’, ‘Dyspareunia’, ‘Vaginal pain’, and ‘Itching’. The symptom ‘Itching’ was recognized by nearly all participants (97.6%, 367 individuals), while ‘Thick vaginal discharge’ was noted by 69.1% (260 individuals). ‘Vaginal pain’ was identified by 29.5% (111 individuals), and ‘Dyspareunia’ was reported by 17.6% (66 individuals). On the other hand, although ‘Fishy odor’ is not a characteristic symptom of VVC, it was mentioned by 41.8% (157 individuals) (Table 5). Only 20 individuals (5.30%) of the participants identified all four correct options, regarding the characteristic symptoms of VVC (Figure 2A).

Comparing the number of correctly identified VVC symptoms according to sociodemographic factors, a weak positive correlation ($r_{S}$: −0.150) was observed between this variable and the number of years of professional experience (Table S1).

In the context of identifying OTC treatments for uncomplicated VVC, clotrimazole vaginal cream 10 mg/g emerged as the most widely recognized option, with 79.5% (299 participants) identifying it. The other topical clotrimazole formulation, including 500 mg vaginal pills and econazole vaginal cream 10 mg/g, were also commonly recognized compared to other medications available in Portuguese pharmacies containing different active ingredients. On the other hand, the econazole vaginal cream + vaginal tablet 10 mg/g + 150 mg was incorrectly selected by more than 60% (230 individuals), and the fenticonazole vaginal soft capsule 600 mg was selected by 25.3% (95 individuals). Each of the remaining options was selected by less than 4% of the participants (Table 6).

The maximum number of correct choices of suitable treatment hypotheses for VVC using OTC medicines (seven) was identified by six participants (1.60%). The identification of less than half of the drugs (zero to three correct answers) was carried out by 255 participants (67.9%) (Figure 2B).

No significant variations in the number of correct responses were observed across the sociodemographic factors analyzed (Table S1).

When asked to distinguish between complicated and uncomplicated VVC based on annual episode count, 22.9% of professionals (n = 86) correctly chose “≥4” (Table 7).

**Figure 3:**
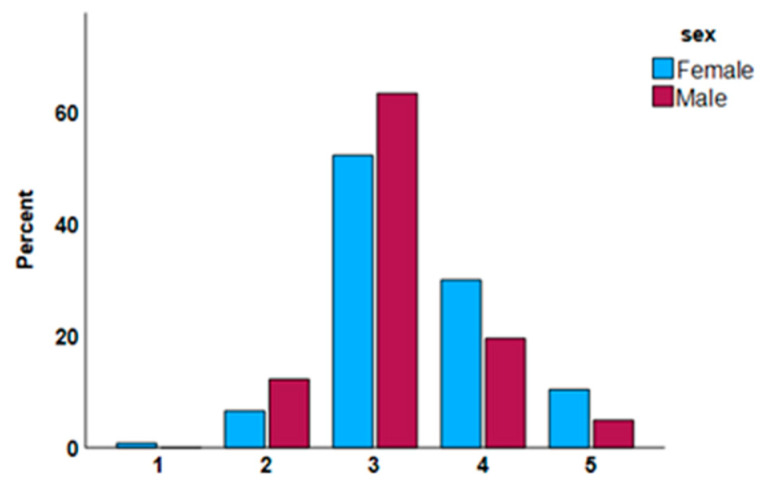
Minimum number of VVC episodes within the same year required to be considered complicated, by sex.

The minimum number of symptoms required to classify VVC as complicated differed based on sex (*p* = 0.038) (Table S1). Among females, 30.0% identified the correct option, whereas less than 20% of males did so. No differences were detected for the other sociodemographic variables (Figure 3).

Pharmacy professionals most commonly recognized fluconazole capsules 150 mg as a treatment for complicated VVC. However, less than half of the respondents (47.9%, 180 individuals) correctly identified this fluconazole formulation as a potential treatment for complicated VVC. The second most identified correct option was the fluconazole 200 mg capsule, although identified by just over 20% (78 individuals). All other correct options were identified by less than 20%. The two most frequently mentioned incorrect options were itraconazole vaginal capsule 100 mg and econazole vaginal cream + vaginal tablet 10 mg/g + 150 mg, each with less than 15% of the responses. A small portion of participants (9.80%, 37 individuals) acknowledged their uncertainty by indicating they were unsure how to respond to this question (Table 8).

Among the various possible treatment options for complicated VVC provided to professionals in the pre-defined answer options, there were 15 that are actually indicated for this purpose. No participant identified the maximum number of correct answers, and the identification of less than half of the drugs for complicated VVC (zero to seven correct answers) was carried out by 364 participants (96.9%) (Figure 2C).

No significant differences were observed in the number of correct responses across the sociodemographic factors analyzed (Table S1).

## 4. Discussion

Several studies have highlighted the importance of assessing healthcare professionals’ knowledge and practices regarding specific health conditions [34,35,36,37]. This study assessed the knowledge of 376 pharmacy professionals regarding VVC and its treatment. In the present study, the greater participation of women compared to men is evident. This discrepancy mirrors the Portuguese healthcare landscape and is consistent with patterns observed in other Western countries, stating that women represent 75% of the healthcare professionals [38].

A review of studies published between 1966 and 2003 revealed that bacterial vaginosis was diagnosed in 22 to 50% of women presenting with symptoms, VVC in 17 to 39%, and trichomoniasis in 4 to 35% [3]. In line with the findings of our study, research analyzing vaginal samples from Portuguese women identified VVC as the leading cause of vaginitis. These results underscore the importance of tailoring treatment and management strategies to the specific needs of the Portuguese population [4]. Consistent with the findings reported by the majority of participants in this study, earlier research conducted between 1997 and 2007, which involved the identification of *Candida* species in isolates collected from medical facilities around the globe, demonstrated that *C. albicans* was the most commonly identified species [39].

Among the characteristic signs and symptoms of VVC, four typical ones were selected for inclusion in the response options provided to participants: itching, thick vaginal discharge, dyspareunia, and vaginal pain. Almost all participants (n = 367, 97.6%) selected itching, which is indeed a major symptom of VVC and even has the potential for differential diagnosis from other causes of vaginitis. In fact, the absence of itching makes the diagnosis of VVC unlikely, as previous studies have shown likelihood ratios (LRs) between 0.18 and 0.79, with a 95% confidence interval, for the association of “absence of itching” and VVC [3]. The second most frequently reported symptom was thick vaginal discharge (n = 260, 69.1%). This is also a characteristic symptom of VVC and, like itching, it is more common in VVC than in other infections such as bacterial vaginosis and trichomoniasis [33,40]. Although only 111 participants selected vaginal pain as a characteristic symptom of VVC, it is important to note that the presence of inflammatory signs is more commonly associated with VVC (LR between 2.1 and 8.4, 95% confidence interval (CI)) [3]. While 157 professionals (41.8%) incorrectly reported a fishy odor as a typical sign of VVC, this is not supported by current medical knowledge. Studies have shown an association between the absence of a fishy odor and VVC among symptomatic women with vaginitis (LR of 2.9, 95% CI) [3]. Conversely, a fishy odor is a hallmark symptom of bacterial vaginosis. Misdiagnosis of these conditions can lead to inappropriate treatment. This finding suggests that pharmacy professionals may have limited knowledge about the specific symptoms of VVC, potentially resulting in incorrect pharmacological recommendations and contributing to the development of antimicrobial resistance [41]. Studies have also shown that women often confuse VVC symptoms with other vaginal infections, hindering accurate diagnosis [27,28,29]. An American study found that only one-third of women, who self-diagnosed and treated VVC with OTC medication (n = 95), actually had the condition [29].

While 22.9% of professionals (86 individuals) correctly identified the minimum number of VVC episodes per year that constitutes a “complicated” case, a substantial portion (57.2%, 215 respondents) provided an incorrect threshold. Additionally, 19.4% of participants admitted they were unsure about the correct answer. This distribution of responses suggests a notable knowledge gap or confusion among healthcare professionals regarding the criteria for classifying VVC cases as “complicated.”. Recognizing complicated cases, such as recurrent VVC (four or more episodes annually), is crucial. This condition affects an estimated 5% to 23% of women and warrants a distinct therapeutic approach [4,33,42,43,44]. Complicated cases may involve non-*albicans* candidiasis and are often more challenging to treat, necessitating intensive therapeutic regimens [6,33].

Recent findings suggest that Portuguese women who use OTC antifungals face a significantly higher risk of developing VVC compared to those who use antifungals prescribed by healthcare professionals [4]. According to guidelines from organizations such as the Infectious Diseases Society of America, topical antifungal agents are recommended for the treatment of uncomplicated VVC, with no single agent superior to others [45]. However, of the seven correct treatment options for uncomplicated VVC presented to the participants (among other incorrect options), 255 participants (67.9%) selected fewer than four of these seven alternatives. Topical formulations containing azoles such as clotrimazole, econazole, miconazol, and fenticonazole are available as OTC medications, facilitating access [46]. Clotrimazole was the most frequently identified drug by participants as suitable for the treatment of uncomplicated VVC in this study, across various presentations. This suggests a higher familiarity among respondents with clotrimazole-based products for treating VVC compared to alternative treatments. Conversely, miconazole 20 mg/g cream was the least identified formulation by pharmacy professionals (n = 13; 3.50%). Respondents with 1 to 5 years of professional experience demonstrated a higher likelihood of providing 4 to 7 correct answers compared to their counterparts in other experience groups. This higher performance may be attributed to the combination of recent academic training in Pharmacy and Pharmaceutical Sciences and a short but substantial period of practical experience. The relatively fresh theoretical knowledge from their studies, coupled with some real-world application, appears to have contributed to their more accurate responses to this particular question. Recent findings indicating a 41% incidence of non-*albicans* candidiasis suggest a much higher incidence of complicated cases among Portuguese women today [4], compared to systematic reviews that revealed a 5–15% incidence of non-*albicans* candidiasis in the 1980s–2000s [2]. The use of inappropriate OTC treatments among this population group therefore takes on an even more concerning dimension and reinforces the importance of in-depth knowledge among pharmacy professionals regarding the best treatment to be instituted. As many pharmacy professionals acknowledged, fluconazole is particularly indicated for complicated VVC cases. For these cases, a 10–14 day induction therapy with oral fluconazole, followed by 150 mg of fluconazole weekly for 6 months, is typically prescribed [45]. Despite being the most frequently identified treatment, less than half of the respondents correctly identified fluconazole as a potential treatment for complicated VVC. This finding is noteworthy, considering that fluconazole is often recommended for treating VVC, especially in its complicated form. The low recognition rate among pharmacy professionals highlights a potential gap in knowledge about current treatment recommendations for complicated VVC. Additionally, although less frequently identified in the present study, topical agents are also considered for induction therapy, in VVC. A review of suitable topical compounds for recurrent VVC identified clotrimazole, miconazole, terconazole, and boric acid as first-line options [47]. Of the 16 correct treatment options for complicated VVC presented to the participants in this study, only 12 out of 376 (3.20%) identified at least half of them.

This is the first study evaluating the knowledge of pharmacy professionals regarding VVC’s main symptoms and correct treatment options. Accordingly, this study could make a significant contribution to identifying the need for further training of these professionals—pharmacists and pharmacy technicians—regarding the approach to be taken in potential VVC cases they may encounter. Additionally, it is worth noting that this study focuses on the assessment of knowledge regarding recommendations established more than a decade ago [33], thus being temporally distant enough from the time of drafting and publication of the guidelines for educational institutions to have adopted them in their curricula. Pharmacy and pharmaceutical sciences courses should adopt effective educational strategies in this area, promoting the correct diagnosis and rational use of antifungal drugs. Some recognized strategies in the context of undergraduate courses of future pharmacy professionals aim at integrated education, which consists of teaching various domains of a subject simultaneously to increase learning effectiveness and student participation [48,49]. The integration of knowledge from different areas of expertise is relevant in the case of VVC, since it requires knowledge from the fields of mycology, pathology, anatomy–physiology, pharmacology, and pharmacotherapy. Methodologies such as case-based learning and e-learning have been effectively used to promote integrated education [50]. However, education cannot be limited to undergraduates. Continuing professional development (CPD) is a key strategy for advancing and optimizing pharmacy practice, contributing to its success [51,52]. Factors associated with positive attitudes about CPD include positive support in the workplace, access to resources and ways to meet learning needs, confidence in the CPD process, and motivation to participate in CPD. Accordingly, both employees and employers have a crucial role in promoting CPD [53].

By revealing some knowledge gaps, this study can serve as a warning to the professionals who consult it, thus enabling them to become aware of the correct information and the best available options. The confusion of other pathologies with VVC can result in an abusive use of the available antifungals as OTC medicine. Although they are usually well tolerated, it should be recognized that topically used antifungals can result in adverse effects such as dyspareunia and irritation, and in some cases, they are presented in the form of oily formulations that increase the porosity of condoms, decreasing their effectiveness [54,55]. In the case of oral antifungals, headache, abdominal pain, nausea, and diarrhea are recognized as common adverse effects [22,54,55]. Besides these potential unnecessary negative impacts of the use of antifungals, the main problem of the wrong diagnosis of any of the main causes of vaginitis is the deprivation of access to the appropriate treatment. Wrong diagnosis and/or wrong choice of drug therapy reduces the probability of solving the problem that led the patient to the pharmacy. Regarding VVC, in severe untreated cases it may cause extensive vulvar erythema, edema, excoriation, and vaginal or vulvar fissures [22]. On the other hand, misinterpreting bacterial vaginosis as VVC might promote bacterial vaginosis’ complications such as endometritis, salpingitis and pelvic inflammatory disease [56]. Finally, one cannot ignore the high global economic burden associated with vaginal infectious diseases caused by direct costs of treatment and indirectly by the loss of productivity, that may persist if diagnosis and/or treatment are incorrect [57,58,59,60].

In terms of methodology, the number of participants in this study is noteworthy. A recent systematic review identified seven studies that assessed pharmacists’ knowledge of Medication Therapy Management. Only one of these studies exceeded the number of participants recruited for the present study (820 participants), while the remaining studies had between 16 and 250 participants [61]. In the present study, a ’snowball’ sampling technique was employed, with initial surveyees forwarding the questionnaires to other interested respondents, thereby maximizing the sample size. This is considered one of the most frequent sampling techniques, and it has a greater potential reach than, for example, sending the questionnaire by email. However, compared to sending emails, this technique has the limitation of not allowing the verification of the response rate among potential participants who were aware of the questionnaire [62]. A common limitation to all online surveys is the fact that they might be completed only by persons who are sufficiently biased to be interested in the subject [63]. The potential selection bias caused by the questionnaire’s dissemination strategy cannot also be disregarded, because the dissemination through social media does not reach all pharmacy professionals. For example, younger age and fewer years of professional experience are positive predictors of use of social media [64], which might have influenced the sample of participants that responded to this questionnaire. Previous studies have already reported that experienced pharmacists are more absent from social media and may have been under-represented in this study, which constitutes an important limitation [65]. Still, social media was the preferred vehicle for the dissemination of the questionnaire in this study, given its cost-effectiveness and potential reach [66]. Social media use worldwide has grown in importance and prevalence, with its estimated number of users at 4.9 billion worldwide [67]. A growing trend is the professional use of social media by healthcare professionals, including those working in a pharmacy setting, motivated by a wide range of factors. Despite its frequent use for chatting, uploading pictures, and keeping in touch with friends, social media has become crucial for healthcare, medical education, and research, promoting collaboration and disseminating findings. More than a decade ago, in a study involving 50 pharmacists from West Virginia (USA), more than 75% of the participants declared to use social media, with Facebook being one of the preferred platforms [68]. In another study conducted in 2014 in Alberta (Canada), more than 80% of the 273 pharmacists that participated in the study assumed to have at least one social media account, with Facebook being already the most frequently used platform [64]. After Facebook (with 2.9 billion monthly active users), Instagram is the second most popular social media platform, with 2 billion monthly active users. As to LinkedIn, it is probably the most popular promotor of professional networking in social media [69]. The reach potential of LinkedIn among pharmacy professionals has already been the subject of characterization in previously published studies [65]. There are no official data on the number of pharmacy technicians; however, according to the National Statistics Institute, in 2023, there were 16,855 pharmacists in Portugal [70]. LinkedIn had about 14,000 profiles registered as pharmacists, in Portugal, in 2025. These data suggest a high representativeness of pharmacists on the social media platform (around 83%). Naturally, this does not mean that everyone had access to the questionnaire through the snowball strategy, but the platform’s potential to be used as a vehicle for disseminating information is high. For the sake of curiosity, there are also 6100 profiles registered on the platform as pharmacy technicians, in Portugal. Given the reach and characteristics of these social media platforms, it was decided in this study to disseminate the questionnaire on these three platforms as measures to promote representativeness. It was already highlighted that the simultaneous use of Facebook and LinkedIn for the dissemination of questionnaires in health professionals promotes fast recruitment, cost-efficiency, snowballing effects, and accessibility of the researcher to potential participants, as the main advantages. On the other hand, it was also concluded that each platform has certain profile tendency, with characteristic sociodemographic features (age, gender, and education level). This fact undoubtedly increases the selection bias [71]. By using the combination of different social media platforms, the authors aimed at increasing representativeness.

Another potential limitation of this study is related to self-reporting bias. Most of the questionnaire questions assess specific technical–scientific knowledge; however, the possibility of self-report bias cannot be excluded in the case of sociodemographic questions such as professional experience [72,73].

Although hospital pharmacists validate all prescriptions issued in the inpatient setting in Portuguese hospitals (including the treatment of VVC, with prescription or OTC medications), one of the limitations of this study is the lack of evaluation of the impact of the area of practice (community or hospital pharmacy). Future studies aiming at characterizing the knowledge of pharmacy professionals may benefit in terms of subgroup analysis based on the characterization of the professionals’ area of practice. It would be valuable to assess the potential impact of routine practice on the knowledge presented regarding specific areas of knowledge, which can only be assessed if researchers question the area of practice of the participants. This variable was not assessed in the present study. The absence of official data on the total number of pharmacy technicians in Portugal precluded the calculation of an adequate sample size. Consequently, the representativeness of the sample cannot be definitively established and is another limitation of this study. Lastly, this study does not explore the reasons behind knowledge gaps or misconceptions, which could be valuable for developing targeted educational interventions. Future studies might explore these reasons and provide valuable information for bachelor and master curricula.

## 5. Conclusions

VVC is a pathology with a high prevalence whose treatment ideally relies on the selection of OTC pharmaceutical products, thus avoiding medical consultations and alleviating pressure on health services. However, based on the results observed in this study, it is concluded that several aspects, related to the correct identification of the pathology (signs and symptoms) and to the treatments available for uncomplicated and complicated VVC, are not known by most pharmacy professionals. The inadequate identification of the pathology, confusing it with other causes of vaginitis and/or the inadequate selection of medications to be dispensed, can compromise therapeutic success, limit patients’ quality of life, increase direct and indirect healthcare costs, and generate mistrust in the community regarding the preparedness of pharmacy professionals. Since continuing education opportunities, whether provided by employers or chosen independently by pharmacists and pharmacy technicians, should focus on areas that demand greater professional engagement and expertise, it is recommended that those professionals attend postgraduate training courses to increase their knowledge in this area, thus promoting the provision of a better service to the community.

## Figures and Tables

**Table 1:** Sociodemographic characteristics of the participants, regarding sex, professional experience, higher academic degree, and profession (*N* = 376).

Sociodemographic Characteristics		n	%
Sex	Female	325	86.4
	Male	51	13.6
Professional Experience	<1 year	47	12.5
	1–5 years	155	41.2
	6–10 years	82	21.8
	11–15 years	48	12.8
	16–20 years	20	5.30
	>21 years	24	6.40
Higher Academic Degree	Bachelor’s Degree	127	33.8
	Master/PhD	249	66.2
Profession	Pharmacist	248	66.0
	Pharmacy Technician	128	34.0

**Table 2:** Main cause of vaginitis identified by pharmacy professionals according to social demographic factors (N = 376). Pairwise comparison is provided using superscript letters. If a pair in a column has any subscript letter in common, then the difference is not significant at a 0.05 level.

	VVC	Bacterial Vaginosis	Trichomoniasis	Other	Does Not Know	*p*-Value
Sex						0.787
Female	184 (56.6%) ^a^	116 (35.7%) ^a^	7 (2.20%) ^a^	5 (1.50%) ^a^	13 (4.00%) ^a^	
Male	33 (64.7%) ^a^	15 (29.4%) ^a^	1 (2.00%) ^a^	1 (2.00%) ^a^	1 (2.00%) ^a^	
Length of professional experience						0.138
<1 year	24 (51.1%) ^a^	19 (40.4%) ^a^	3 (6.40%) ^a^	0 (0.00%) ^a^	1 (2.10%) ^a^	
1–5 years	79 (51.0%) ^a^	61 (30.4%) ^a^	1 (0.60%) ^a^	4 (2.60%) ^a^	10 (6.5%) ^a^	
6–10 years	53 (64.6%) ^a^	24 (29.3%) ^a^	3 (3.70%) ^a^	1 (1.20%) ^a^	1 (1.20%) ^a^	
11–15 years	34 (70.8%) ^a^	13 (27.1%) ^a^	0 (0.00%) ^a^	0 (0.00%) ^a^	1 (2.10%) ^a^	
16–20 years	14 (70.0%) ^a^	4 (20.0%) ^a^	1 (5.00%) ^a^	1 (5.0%) ^a^	0 (0.00%) ^a^	
>21 years	13 (54.2%) ^a^	10 (41.7%) ^a^	0 (0.00%) ^a^	0 (0.00%) ^a^	1 (4.20%) ^a^	
High Academic Degree						0.380
Bachelor	68 (53.5%) ^a^	50 (39.4%) ^a^	2 (1.60%) ^a^	2 (1.60%) ^a^	5 (3.90%) ^a^	
Master/PhD	149 (59.8%) ^a^	81 (32.5%) ^a^	6 (2.40%) ^a^	4 (1.60%) ^a^	9 (3.60%) ^a^	
Profession						0.677
Pharmacist	148 (59.7%) ^a^	84 (33.9%) ^a^	6 (2.40%) ^a^	3 (1.20%) ^a^	7 (2.80%) ^a^	
Pharmacy Technician	69 (53.9%) ^a^	47 (36.7%) ^a^	2 (1.60%) ^a^	3 (2.30%) ^a^	7 (5.50%) ^a^	
Total	217 (57.7%)	131 (34.8%)	8 (2.13%)	6 (1.60%)	14 (3.72%)	−

**Table 3:** Main etiological agent of VVC identified by pharmacy professionals according to social demographic factors (N = 376). Pairwise comparison is provided using superscript letters “a” and “b”. If a pair in a column has any superscript letter in common, then the difference is not significant at a 0.05 level.

	*Candida albicans*	*Candida glabrata*	*Candida tropicalis*	Does Not Know	Prefers Not to Answers	*p*-Value
Sex						0.021
Female	313 (96.3%) ^a^	3 (0.90%) ^a^	2 (0.60%) ^a^	6 (1.80%) ^a^	1 (0.30%) ^a^	
Male	45 (88.2%) ^b^	4 (7.80%) ^b^	0 (0.00%) ^a^	2 (3.90%) ^a^	0 (0.00%) ^a^	
Length of professional experience						0.481
<1 year	43 (91.5%) ^a^	1 (2.10%) ^a^	1 (2.10%) ^a^	1(2.10%) ^a^	1 (2.10%) ^a^	
1–5 years	148 (95.5%) ^a^	2 (1.30%) ^a^	0 (0.00%) ^a^	5 (3.20%) ^a^	0 (0.00%) ^a^	
6–10 years	80 (97.6%) ^a^	1 (1.20%) ^a^	0 (0.00%) ^a^	1 (1.20%) ^a^	0 (0.00%) ^a^	
11–15 years	44 (91.7%) ^a^	2 (4.20%) ^a^	1 (2.10%) ^a^	1 (2.10%) ^a^	0 (0.00%) ^a^	
16–20 years	19 (95.0%) ^a^	1 (5.00%) ^a^	0 (0.00%) ^a^	0 (0.00%) ^a^	0 (0.00%) ^a^	
>21 years	24 (100%) ^a^	0 (0.00%) ^a^	0 (0.00%) ^a^	0 (0.00%) ^a^	0 (0.00%) ^a^	
High Academic Degree						0.394
Bachelor	118 (92.9%) ^a^	3 (2.40%) ^a^	1 (0.80%) ^a^	4 (3.10%) ^a^	1 (0.80%) ^a^	
Master/PhD	240 (96.4%) ^a^	4 (1.60%) ^a^	1 (0.40%) ^a^	4 (1.60%) ^a^	0 (0.00%) ^a^	
Profession						0.048
Pharmacist	241 (97.2%) ^a^	2 (0.80%) ^a^	1 (0.40%) ^a^	4 (1.60%) ^a^	0 (0.00%) ^a^	
Pharmacy Technician	117 (91.4%) ^b^	5 (3.90%) ^b^	1 (0.80%) ^a^	4 (3.10%) ^a^	1 (0.80%) ^a^	
Total	358 (95.2%)	7 (1.90%)	2 (0.50%)	8 (2.10%)	1 (0.30%)	−

**Table 4:** Logistic regression model for the identification of *Candida albicans* as the correct main etiological agent of VVC.

	N	OR	95% CI	*p*-Value
Sex				
Female	318	−	−	−
Male	49	0.129	0.029–0.569	0.007
Length of professional experience				
<1 year	45	−	−	−
1–5 years	150	4.57	0.57–36.7	0.150
6–10 years	81	8.40	0.49–143	0.140
11–15 years	47	0.87	0.12–6.13	0.890
16–20 years	20	1.50	0.10–23.2	0.770
>20 years	24	^a^	^a^	^a^
High Academic Degree				
Bachelor	122	−	−	−
Master/PhD	245	0.171	0.023–1.279	0.085
Profession				
Pharmacist	244	−	−	−
Pharmacy Technician	123	0.048	0.006–0.404	0.005

^a^ No incorrect answers were observed.

**Table 5:** Number and percentage of professionals that correctly identified each of the main VVC symptoms (N = 376).

	Symptoms	N	%
Correct symptoms	Itching	367	97.6
	Thick vaginal discharge	260	69.1
	Vaginal pain	111	29.5
	Dyspareunia	66	17.6
Incorrect symptoms	Fishy odor	157	41.8
	Vaginal dryness	46	12.2
	Foamy vaginal discharge	36	9.60
	Punctate hemorrhages (strawberry cervix)	10	2.70
	Postcoital bleeding	9	2.40
	Other	2	0.50

**Table 6:** Absolute frequency and percentage of participants who chose each of the correct over-the-counter medicine options in the pre-defined answer options, for the treatment of uncomplicated VVC (N = 376).

	Active Ingredient, Formulation, Concentration	n	%
Correct medicine options	Clotrimazole, vaginal cream, 10 mg/g	299	79.5
	Clotrimazole, vaginal pill, 500 mg	194	51.6
	Econazole, vaginal cream, 10 mg/g	149	39.6
	Clotrimazole, vaginal pill, 100 mg	148	39.4
	Econazole, vaginal tablet, 150 mg	116	30.9
	Fenticonazole, vaginal tablet, 200 mg	79	21.0
	Miconazole, cream, 20 mg/g	13	3.50
Incorrect medicine options	Econazole, vaginal cream + vaginal tablet, (10 mg/g) + (150 mg)	230	61.2
	Fenticonazole, vaginal soft capsule, 600 mg	95	25.3
	Fenticonazole, vaginal cream, 20 mg/g	13	3.50
	Fluconazole, vaginal capsule, 150 m	8	2.10
	Sertaconazole, vaginal tablet, 300 mg	7	1.90
	Isoconazole, vaginal cream, 10 mg/mL	6	1.60
	Dequalinium chloride, vaginal pill, 10 mg	5	1.30
	Fluconazole, vaginal capsule, 200 mg	5	1.30
	Sertaconazole, vaginal cream, 20 mg/g	5	1.30
	Fluconazole, vaginal capsule, 50 mg	4	1.10
	Fosfomycin, granules for oral solution, 2000 mg	3	0.80
	Fosfomycin, granules for oral solution, 3000 mg	3	0.80
	Metronidazole, vaginal tablet, 500 mg	3	0.80
	Clindamycin, vaginal cream, 20 mg/g	2	0.50
	Itraconazole, vaginal capsule, 100 mg	2	0.50
	Itraconazole, oral solution, 10 mg/ml	2	0.50
	Nifuratel + Nystatin, vaginal tablet, 500 mg + 200,000 I.U.	2	0.50
	Nystatin, oral suspension, 100,000 I.U./mL	2	0.50
	Sertaconazole, vaginal tablet, 500 mg	2	0.50
	Fluconazole, powder for oral suspension, 40 mg/mL	0	0.00
	Does not know	3	0.80
	Prefers not to answer	2	0.50

**Table 7:** Number of VVC episodes in the same year from which it is considered complicated, according to pharmacy professionals (N = 376).

Number of Episodes	N	%
≥1	2	0.50
≥2	22	5.90
≥3	162	43.1
≥4	86	22.9
≥5	29	7.70
Does not know	73	19.4
Prefers not to answer	2	0.50

**Table 8:** Absolute frequency and percentage of participants who chose each of the correct medicine options in the pre-defined answer options, for the treatment of complicated VVC (N = 376).

	Active Ingredient, Formulation, Concentration	n	%
Correct medicine options	Fluconazole, capsule, 150 mg	180	47.9
	Fluconazole, capsule, 200 mg	78	20.7
	Miconazole, cream, 20 mg/g	74	19.7
	Sertaconazole, vaginal cream, 20 mg/g	65	17.3
	Fluconazole, capsule, 50 mg	60	16.0
	Sertaconazole, vaginal capsule, 300 mg	48	12.8
	Sertaconazole, vaginal capsule, 500 mg	45	12.0
	Clotrimazole, vaginal cream, 10 mg/g	43	11.4
	Fenticonazole, vaginal tablet, 200 mg	41	10.9
	Fenticonazole, vaginal cream, 20 mg/g	34	9.00
	Clotrimazole, vaginal pill, 500 mg	33	9.40
	Isoconazole, vaginal cream, 10 mg/g	31	8.20
	Clotrimazole, vaginal pill, 100 mg	21	5.60
	Econazole, vaginal cream, 10 mg/g	21	5.60
	Econazole, vaginal capsule, 150 mg	20	5.30
Incorrect medicine options	Itraconazole, vaginal capsule, 100 mg	56	14.9
	Econazole, vaginal cream + vaginal tablet, (10 mg/g) + (150 mg)	54	14.4
	Clindamycin, vaginal cream, 20 mg/g	41	10.9
	Dequalinium chloride, vaginal pill, 10 mg	39	10.4
	Fenticonazole, vaginal soft capsule, 600 mg	35	9.30
	Nifuratel + Nystatin, vaginal tablet, 500 mg + 200,000 I.U.	17	4.50
	Itraconazole, oral solution, 10 mg/mL	16	4.30
	Fluconazole, powder for oral suspension, 40 mg/mL	14	3.70
	Metronidazole, vaginal tablet, 500 mg	9	2.40
	Fosfomycin, granules for oral solution, 3000 mg	6	1.60
	Fosfomycin, granules for oral solution, 2000 mg	4	1.10
	Nystatin, oral suspension, 100,000 I.U./mL	2	0.50
	None	2	0.50
	Does not know	37	9.80
	Prefers not to answer	17	4.50

## Data Availability

The original contributions presented in this study are included in the article/Supplementary Materials. Further inquiries can be directed to the corresponding author(s).

## Supplementary Materials

The following supporting information can be downloaded at https://www.mdpi.com/article/10.3390/healthcare13040402/s1: Table S1: Number of correct options identified by pharmacy professionals according to social demographic factors. Figure S1: Original version of the submitted questionnaire.
