# Supplementary material for: Knowledge of Vulvovaginal Candidiasis Characteristics, Signs, Symptoms, and Appropriate Treatment Among Portuguese Pharmacy Professionals
Source: Healthcare (Basel). 2025 Feb 13;13(4):402. doi: 10.3390/healthcare13040402 (PMC11855004; doi:10.3390/healthcare13040402)
Supplement: Supplementary file 1 [file healthcare-13-00402-s001.zip › healthcare-3463605-supplementary/Supplementary_Material Figure S1.pdf]

O Uso Racional de Antifúngicos para Infeções Vaginais por Profissionais de Farmácia

* Obrigatória

Consentimento Informado

Os objetivos do estudo são (i) a caracterização dos conhecimentos dos profissionais de farmácia quanto a sinais e sintomas de candidíase vulvovaginal e quanto às diferenças entre candidíase vulvovaginal complicada e não complicada; (ii) a caracterização dos conhecimentos dos profissionais de farmácia (farmacêuticos, técnicos superiores de farmácia e técnicos auxiliares de farmácia) quanto aos tratamentos disponíveis para candidíase vulvovaginal não complicada, e quanto a práticas que podem diminuir a sua eficácia.

Nesse sentido, convidamos-vos a responder ao questionário retratando os vossos conhecimentos e a forma como os aplicam/aplicariam em contexto real, no âmbito do normal desempenho das vossas funções.

Os investigadores garantem aos participantes que todo o questionário é confidencial e as respostas serão mantidas no anonimato. Os potenciais participantes podem recusar-se a participar ou interromper a qualquer momento a participação no estudo, sem nenhum tipo de penalização, por esse facto.

Se pretender que lhe seja esclarecida qualquer questão antes da potencial participação, durante as respostas ao questionário, ou após a conclusão do questionário, poderá fazê-lo através do seguinte e-mail: e-mail: 10180527@ess.ipp.pt

Seguidamente, ao clicar em "Prosseguir" consente com a recolha dos seus dados para posterior tratamento e análise estatística. Se não quiser prosseguir e/ou não consentir com a recolha de dados e com a participação terá de selecionar a opção "Desistir".

Ao clicar em "Prosseguir" declara igualmente que os dados sejam alvo de publicação em qualquer formato e forma de exibição ou divulgação incluindo em trabalhos imprimidos, eletrónicos e/ou em versões digitais disponíveis na *world wide web*, sempre sendo salvaguardado o anonimato enquanto participante e não sendo exibidos quaisquer informações pessoais relevantes de forma isolada que permitam a sua identificação enquanto participante.

1. *

◯ Prosseguir

◯ Desistir

Informações Demográficas

2. Género *

- ◯ Feminino
- ◯ Masculino
- ◯ Não responde

3. Indique o tempo de experiência profissional que possuía, em outubro de 2023. *

4. Qual a habilitação máxima que possui? *

- ◯ Bacharelato
- ◯ Licenciatura
- ◯ Mestrado
- ◯ Doutoramento
- ◯ Outra
- ◯ Não responde

5. Qual? *

6. Qual a sua profissão? *

- ◯ Farmacêutico/a
- ◯ Técnico/a de Farmácia
- ◯ Técnico/a Auxiliar de Farmácia
- ◯ Outro

7. Qual o distrito onde desempenha funções, ou, em caso de pausa na carreira, onde desempenhou funções mais recentemente? (em caso de desempenho em diferentes locais, indique o distrito onde desempenha funções, durante mais tempo)
*

- ◯ Aveiro
- ◯ Beja
- ◯ Braga
- ◯ Bragança
- ◯ Castelo Branco
- ◯ Coimbra
- ◯ Évora
- ◯ Faro
- ◯ Guarda
- ◯ Leiria
- ◯ Lisboa
- ◯ Portalegre
- ◯ Porto
- ◯ Região Autónoma da Madeira
- ◯ Região Autónoma dos Açores
- ◯ Santarém
- ◯ Setúbal
- ◯ Viana do Castelo
- ◯ Vila Real
- ◯ Viseu

Questionário

8. Qual a principal causa de vaginite? *

- ◯ Vaginose bacteriana
- ◯ Tricomoníase
- ◯ Candidíase vulvovaginal
- ◯ Nenhum
- ◯ Não sabe
- ◯ Não responde

9. Qual o principal agente etiológico da candidíase vulvovaginal? *

- ◯ *Candida glabrata*
- ◯ *Candida albicans*
- ◯ *Candida tropicalis*
- ◯ Nenhum
- ◯ Não sabe
- ◯ Não responde

10. Qual das seguintes representa fator de risco para o desenvolvimento de candidíase vulvovaginal? (pode selecionar várias opções) *

- [ ] Gravidez
- [ ] Menopausa
- [ ] Diabetes
- [ ] Hipercolestorelemia
- [ ] Hipertensão
- [ ] Uso de preservativo
- [ ] Uso de anticoncecionais orais
- [ ] Uso de espermicidas
- [ ] Uso de roupas íntimas sintéticas justas
- [ ] Utilização de piscinas públicas
- [ ] Elevada ingestão de açucares refinados
- [ ] Antibiótico
- [ ] Elevada ingestão de produtos hortículas
- [ ] Tabagismo
- [ ] Ingestão de bebidas alcoólicas
- [ ] Nenhum
- [ ] Não sabe
- [ ] Não responde

11. Qual dos seguintes sintomas é sugestivo de candidíase vulvovaginal? (pode selecionar várias opções) *

- [ ] Prurido
- [ ] Dor vaginal
- [ ] Dispareunia
- [ ] Hemorragia pós-coito
- [ ] Secura vaginal
- [ ] Descargas vaginais espumosas
- [ ] Descargas vaginais espessas
- [ ] Odor fétido
- [ ] Hemorragias puntiformes (manchas de morango)
- [ ] Nenhum
- [ ] Não sabe
- [ ] Não responde

12. Admita que diagnostica um caso de candidíase vulvovaginal e a utente não apresenta receita médica. Qual dos seguintes medicamentos escolheria? (pode selecionar várias opções) *

MNSRM - Medicamento não sujeito a receita médica
MNSRM/EF - Medicamento não sujeito a receita médica de dispensa exclusiva em farmácia
MSRM - Medicamento sujeito a receita médica
U.I. - Unidades Internacionais

- [ ] Clindamicina, creme vaginal, 20mg/g (Dalacin V) - MSRM
- [ ] Cloreto de dequalínio, comprimido vaginal, 10mg (Fluomizin) - MSRM
- [ ] Clotrimazol, comprimido vaginal, 100mg (Gino-Canesten) - MNSRM
- [ ] Clotrimazol, comprimido vaginal, 500mg (Gino-Canesten 1) - MNSRM
- [ ] Clotrimazol, creme vaginal, 10mg/g (Gino-Canesten) - MNSRM
- [ ] Econazol, creme vaginal, 10mg/g (Gyno-Pevaryl) - MNSRM
- [ ] Econazol, creme vaginal + óvulo, (10 mg/g) + (150 mg) (Gyno-Pevaryl Combipack) - MNSRM
- [ ] Econazol, óvulo, 150mg (Gyno-Pevaryl) - MNSRM
- [ ] Fenticonazol, cápsula mole vaginal, 600mg (Lomexin) - MNSRM-EF
- [ ] Fenticonazol, creme, 20mg/g (Lomexin) - MSRM
- [ ] Fenticonazol, óvulo, 200mg (Lomexin) - MNSRM-EF
- [ ] Fluconazol, cápsula, 50mg (Diflucan) - MSRM
- [ ] Fluconazol, cápsula, 150mg (Diflucan 150) - MSRM
- [ ] Fluconazol, cápsula, 200mg (Génerico) - MSRM
- [ ] Fluconazol, pó para suspensão oral, 40mg/ml (Diflucan) - MSRM
- [ ] Fosfomicina, granulado para solução oral, 2000 mg (Fosfomicina Monuril) - MSRM
- [ ] Fosfomicina, granulado para solução oral, 3000 mg (Fosfomicina Monuril) - MSRM
- [ ] Isoconazol, creme vaginal, 10mg/ml (Gino-Travogen) - MSRM
- [ ] Itraconazol, cápsula, 100mg (Sporanox) - MSRM
- [ ] Itraconazol, solução oral, 10mg/ml (Sporanox) - MSRM
- [ ] Metronidazol, óvulo, 500mg (Flagyl) - MSRM
- [ ] Miconazol, creme, 20mg/g (Daktarin) - MNSRM
- [ ] Nifuratel + Nistatina, óvulo, 500mg + 200000 U.I. (Dafnegil) - MSRM
- [ ] Nistatina, suspensão oral, 100000 U.I./ml (Mycostatin) -MNSRM
- [ ] Sertaconazol, comprimido vaginal, 500mg (Dermofix) - MSRM
- [ ] Sertaconazol, creme vaginal, 20mg/g (Dermofix) - MSRM
- [ ] Sertaconazol, óvulo, 300mg (Dermofix) - MSRM
- [ ] Nenhum

13. Qual o número de episódios de candidíase, no mesmo ano, a partir do qual se considera a candidíase como complicada? *

- ◯ + de 1
- ◯ + de 2
- ◯ + de 3
- ◯ + de 4
- ◯ + de 5
- ◯ Nenhum
- ◯ Não sabe
- ◯ Não responde

14. Perante candidíase vulvovaginal complicada, qual dos seguintes tratamentos considera que seria recomendável? (pode selecionar várias opções) *

- [ ] Clindamicina, creme vaginal, 20mg/g (Dalacin V) - MSRM
- [ ] Cloreto de dequalínio, comprimido vaginal, 10mg (Fluomizin) - MSRM
- [ ] Clotrimazol, comprimido vaginal, 100mg (Gino-Canesten) - MNSRM
- [ ] Clotrimazol, comprimido vaginal, 500mg (Gino-Canesten 1) - MNSRM
- [ ] Clotrimazol, creme vaginal, 10mg/g (Gino-Canesten) - MNSRM
- [ ] Econazol, creme vaginal, 10mg/g (Gyno-Pevaryl) - MNSRM
- [ ] Econazol, creme vaginal + óvulo, (10 mg/g) + (150 mg) (Gyno-Pevaryl Combipack) - MNSRM
- [ ] Econazol, óvulo, 150mg (Gyno-Pevaryl) - MNSRM
- [ ] Fenticonazol, cápsula mole vaginal, 600mg (Lomexin) - MNSRM-EF
- [ ] Fenticonazol, creme, 20mg/g (Lomexin) - MSRM
- [ ] Fenticonazol, óvulo, 200mg (Lomexin) - MNSRM-EF
- [ ] Fluconazol, cápsula, 50mg (Diflucan) - MSRM
- [ ] Fluconazol, cápsula, 150mg (Diflucan 150) - MSRM
- [ ] Fluconazol, cápsula, 200mg (Génerico) - MSRM
- [ ] Fluconazol, pó para suspensão oral, 40mg/ml (Diflucan) - MSRM
- [ ] Fosfomicina, granulado para solução oral, 2000 mg (Fosfomicina Monuril) - MSRM
- [ ] Fosfomicina, granulado para solução oral, 3000 mg (Fosfomicina Monuril) - MSRM
- [ ] Isoconazol, creme vaginal, 10mg/ml (Gino-Travogen) - MSRM
- [ ] Itraconazol, cápsula, 100mg (Sporanox) - MSRM
- [ ] Itraconazol, solução oral, 10mg/ml (Sporanox) - MSRM
- [ ] Miconazol, creme, 20mg/g (Daktarin) - MNSRM
- [ ] Metronidazol, óvulo, 500mg (Flagyl) - MSRM
- [ ] Nifuratel + Nistatina, óvulo, 500mg + 200000 U.I. (Dafnegil) - MSRM
- [ ] Nistatina, suspensão oral, 100000 U.I./ml (Mycostatin) -MNSRM
- [ ] Sertaconazol, comprimido vaginal, 500mg (Dermofix) - MSRM
- [ ] Sertaconazol, creme vaginal, 20mg/g (Dermofix) - MSRM
- [ ] Sertaconazol, óvulo, 300mg (Dermofix) - MSRM
- [ ] Nenhum
- [ ] Não sabe
- [ ] Não responde

Este conteúdo não foi criado nem é aprovado pela Microsoft. Os dados que submeter serão enviados para o proprietário do formulário.

Microsoft Forms
